# Habitual, perceived ideal and neutral sitting postures within an asymptomatic young adult population: muscle activity and sagittal spinal curvature

**DOI:** 10.1186/1748-7161-8-S2-O37

**Published:** 2013-09-18

**Authors:** Steven Mabb, Josette Bettany-Saltikov, David Hodgson

**Affiliations:** 1 Teesside University, Middlesbrough, UK

## Background

Sitting posture is a common aggravating factor for pain both at the cervical and lumbar spine regions, with extended periods of sitting associated with increased discomfort at the neck and upper and lower back. Sitting postures independently adopted by asymptomatic individuals in comparison to theorized ‘optimal’ sitting postures have not been fully evaluated (O’Sullivan et al., 2010). If a clinically significant imbalance exists between these postures, intervention may be required to optimize the postural habits of the general public. 

## Purpose

The purpose of this study was to quantitatively compare three upright, unsupported sitting postures: the habitual sitting posture (HSP), subjectively perceived ideal posture (SPIP) and neutral sitting posture (NSP). 

## Methods

A convenience sample of 24 asymptomatic young adults participated in this study. Spinal posture was analyzed from C2-L5 using the Microscribe 3DX digitiser. Five muscles were measured by surface electromyography. Differences between postures for spinal curvature were analyzed using ANOVA. Friedman’s ANOVA was used to analyze muscle activity. 

## Results

HSP was more kyphosed than the NSP at the upper lumbar spine region (MD 4.63 with a 95% CI = 1.97 to 7.29). The SPIP was less kyphosed at the lower thoracic spine region (MD of -2.31 with 95% CI = -5.31 to -0.41) compared to the NSP. Muscle activity was greater at the cervical erector spinae for the HSP, compared to both the SPIP (p= 0.001) and NSP (p= 0.001). Muscle activity was greater during the SPIP and NSP, compared to the HSP for the thoracic erector spinae (p< 0.044) and external oblique (p< 0.006); no differences in muscle activity between the SPIP and NSP were identified. 

## Conclusions and discussion

The NSP was identified as a relatively midrange sitting posture compared to HSP and SPIP. Education that aims to optimize asymptomatic individuals’ sitting habits may therefore benefit from highlighting the importance of midrange sitting postures. It should be noted that both the NSP and SPIP, when maintained, will likely lead to increased levels of fatigue due to increased global muscle activity, compared to the HSP. To reduce the onset of fatigue during prolonged periods of unsupported sitting, incorporation of active movements may be beneficial (Pynt, Higgs and Mackey, 2002). However, further study is required to support this. 
